# Transforming financial documents into credit decisions using explainable artificial intelligence and optical character recognition

**DOI:** 10.1016/j.mex.2026.103962

**Published:** 2026-05-18

**Authors:** Sachin Malave, Bharti Khemani, Hrishit Patil, Sanket Nandurkar, Om Nandurkar, Ayush Nayak

**Affiliations:** a Head of Computer Engineering Department, A. P. SHAH Institute of Technology, Survey No 12, 13, Opp. Hypercity Mall, Kasarvadavali, Ghodbunder Road, Thane West, Thane, Maharashtra 400615, India; b Assistant Professor, A. P. SHAH Institute of Technology, Survey No 12, 13, Opp. Hypercity Mall, Kasarvadavali, Ghodbunder Road, Thane West, Thane, Maharashtra 400615, India; c Student, A. P. SHAH Institute of Technology, Survey No 12, 13, Opp. Hypercity Mall, Kasarvadavali, Ghodbunder Road, Thane West, Thane, Maharashtra 400615, India

**Keywords:** Explainable artificial intelligence (XAI), Credit scoring, Optical character recognition (OCR), Financial document processing, Machine learning, XGBoost, SHAP, LIME

## Abstract

The increasing use of digital technology in banking and financial services has accelerated the adoption of Artificial Intelligence and Machine Learning in lending and credit scoring. While advanced ensemble models offer higher predictive accuracy than traditional statistical approaches, they are often difficult to interpret. This lack of transparency raises concerns related to fairness, regulatory compliance, and borrower trust. This study proposes an integrated Explainable Artificial Intelligence framework for automated credit scoring and lending decisions. The framework utilizes Optical Character Recognition through EasyOCR, PyPDF2, and PdfPlumber to extract financial and identity data from applicant documents, followed by data cleaning, normalization, and structured feature engineering. SHAP and LIME are applied to generate global and local explanations, enhancing interpretability. Experimental evaluation shows that XGBoost achieves 92.5% accuracy, outperforming LightGBM (90.8%), Random Forest (87%), Logistic Regression (91%), and LSTM (81%).

• The methodology integrates document-based data extraction with supervised learning models to generate accurate credit scores and lending decisions.
• Performance metrics including accuracy, precision, recall, F1-score, MAE, RMSE, and R² confirm the superior performance of XGBoost in credit risk prediction.
• The results highlight that combining structured financial features with explainable models improves transparency, supports regulatory compliance, and enhances decision-making reliability.

## Graphical abstract

**Figure:**
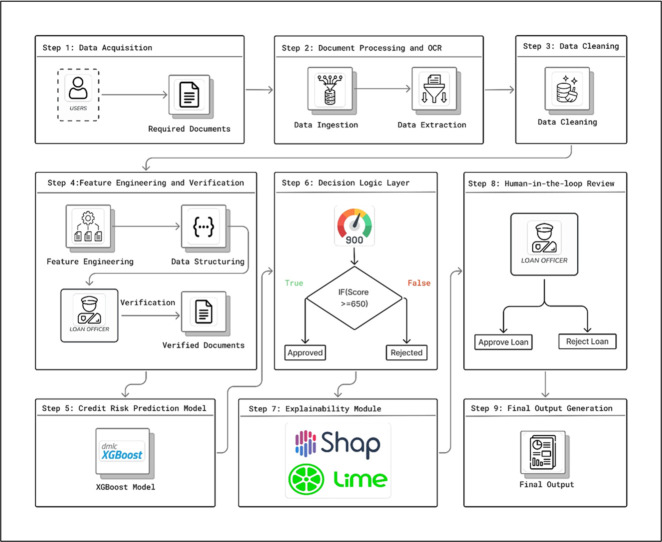

Specifications table

**Table:** 

Subject area	Engineering
More specific subject area	Credit Risk Assessment and Financial Document Analysis
Name of your method	Explainable Artificial Intelligence (XAI), Optical Character Recognition (OCR), XGBoost, Credit Scoring
Name and reference of original method	N/A
Resource availability	https://github.com/Hrishit-Patil/FynXai

## Background

The increasing rate of digitization of banking and financial services has resulted in a rapid increase in the number of automated credit evaluation systems. Credit scoring plays an important role in determining the eligibility of a borrower and mitigating financial risk. However, traditional credit scoring systems employing logistic regression and rule-based systems are limited in effectively managing complex non-linear relationships [1]. Although these systems provide interpretability, these systems are limited in effectively utilizing the variety and dimensionality of data available in modern financial systems. In contrast, Random Forest and Gradient Boosting have shown promising results in credit scoring systems [2,3]. However, these systems are limited in providing interpretability, and fairness and accountability are major concerns [4].

One major drawback in existing credit scoring systems is that these systems are limited to utilizing structured data. In real-world applications, a major portion of applicant data is available in semi-structured or unstructured formats [5]. These data may be in the form of salary slips, bank statements, or identification documents. Although Optical Character Recognition (OCR) and document parsing are employed to extract data from these documents [6], these systems are not effectively employed in credit scoring systems [7,8]. This results in the underutilization of financial data available from documents.

Explainable Artificial Intelligence (XAI) methods like SHAP and LIME have been introduced to deal with the interpretability problems of complex machine learning models. XAI methods offer global and local explanations, facilitating the interpretation of feature contribution [9,10]. However, most studies have been conducted on structured data, and no research has been conducted to incorporate XAI methods with features generated from documents [11].

The motivation behind this methodology is to bridge the existing gap by creating a unified document-centric framework that unifies data extraction, feature engineering, prediction, and decision support using explainable AI techniques [12,13]. The method proposed here will enable the conversion of semi-structured financial documents to structured features, which can be fed to machine learning models, and at the same time, maintain interpretability using XAI techniques [14]. Furthermore, the addition of the human-in-the-loop validation mechanism will ensure the accuracy and compliance of the system.

This methodology offers a practical and efficient solution for real-world credit systems, in which predictive performance and interpretability are critical [15,16]. The proposed approach enables more accurate, transparent, and compliant credit decision-making processes by leveraging the power of document intelligence and machine learning interpretability [17].

### Literature review

Traditionally, credit scoring has been carried out using statistical methods like Logistic Regression along with decision rules, which have interpretability but lack the ability to capture complex non-linear associations in financial data [18]. However, such models fall short in dealing with high-dimensional and heterogenous financial data.

To address these limitations, machine learning models such as Decision Trees, Random Forest, Gradient Boosting, and XGBoost have been widely adopted. These models significantly improve predictive performance by capturing nonlinear relationships and interactions among financial attributes [19]. However, despite their high accuracy, these approaches often function as black-box models, raising concerns related to interpretability, fairness, and regulatory compliance in financial decision-making systems [20].

Simultaneous advances in OCR technology and document processing algorithms have made it possible to retrieve financial information from unstructured and semi-structured data sources, including bank statements and salary receipts [21]. Although the above technologies improve access to data, currently known solutions mainly focus on separate processing of the documents and fail to incorporate the retrieved data into models for predicting credit scores [22].

To improve transparency, Explainable Artificial Intelligence (XAI) techniques such as SHAP and LIME have been introduced, providing both global and local interpretability of model predictions [23,24]. These methods are increasingly applied in financial systems to enhance trust and support regulatory requirements. However, existing research mainly concentrates on structured data and does not include any explainability to extract features from document-based data sources.

There have been efforts made to investigate hybrid approaches that can incorporate both machine learning and explainability techniques through the use of ensemble methods like XGBoost and SHAP [25]. However, there are still difficulties faced when trying to implement the integration of document intelligence, prediction models, and explainability.

Therefore, there remains a clear research gap in developing an end-to-end system that seamlessly integrates document-based feature extraction, machine learning-based credit scoring, and explainable decision-making. The proposed methodology addresses this gap by presenting a unified, document-driven, explainable credit scoring framework.

## Method details

**Fig. 1:**
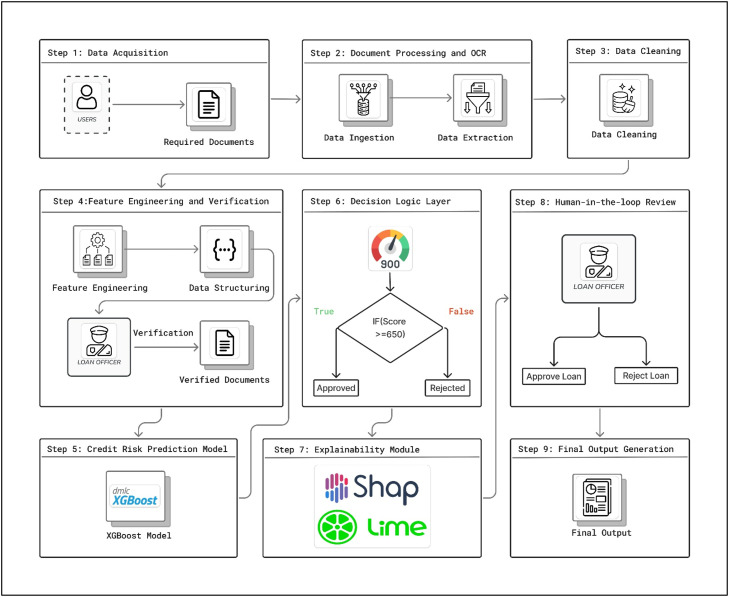
Proposed methodology.

The proposed method provides a novel end-to-end document-driven explainable credit scoring framework that encompasses the entire process of document ingestion, structured data extraction, feature engineering, prediction modeling, and explainable decision support under a single framework, as depicted in Fig. 1. In the first step, various financial and identification documents are provided by the applicant, which are then processed by Optical Character Recognition (OCR) and Structured PDF Parsing techniques to extract relevant information from the documents. The extracted information is then converted into a JSON structure, followed by data cleaning, normalization, and verification of the extracted information from various documents. Next, the feature engineering module converts the extracted information into a standardized structure of 15 features, including financial and behavioral attributes of the applicant's credit history. These features are then verified by a loan officer, followed by a trained Extreme Gradient Boosting (XGBoost) model that predicts a numerical value of the credit score based on the features provided by the feature engineering module. Based on the predicted credit score, the rule-based decision module decides the eligibility of the applicant, the interest rate, and the sanctioned amount of the loan by using EMI constraints. To increase the transparency of the model, SHAP values and LIME values are used to generate global and instance-level explanations of the model's predictions. Finally, the human-in-the-loop concept enables the loan officer to review the results of the model predictions and explanations prior to making a final decision.

### Data acquisition

The proposed system uses two data sources: applicant documents and a structured dataset used for training models.

Applicant Documents: The proposed system requires the applicant to submit four categories of documents, namely the Aadhaar Card, PAN Card, salary slips for the last three months, and bank statements for the last six months. These documents contain semi-structured and unstructured data related to financial and identification information of the applicant. Training Dataset: A publicly available dataset is used to train the model, which is obtained from the Kaggle data science community. This dataset consists of 15 features related to financial and behavioural information of applicants, and one target variable related to credit scores.

### Document processing and OCR extraction

The processing of the document happens with a combination of pdfplumber and PyPDF libraries. After processing, the information is represented in a hierarchical JSON structure. The document types, formats, and corresponding extracted attributes used for downstream processing are specified in Table 1.

**Table 1:** Document Types and Extracted Fields.

Document Type	Format	Key Extracted Fields
Aadhaar Card	Image	Name, DOB, Gender, Address, Aadhaar Number
PAN Card	Image	Name, PAN Number, DOB
Salary Slips	PDF	Net Pay, Gross Salary, Employer, Deductions, Provident Fund
Bank Statement	PDF	Transactions, Balance, EMI, Credit/Debit Summary

The system uses role-based authentication and audit trails to ensure that only authorized personnel have access to critical applicant data. The documents are processed solely for OCR purposes, after which they are deleted to maintain data privacy. Furthermore, the human-in-the-loop feature ensures that all decisions are reviewed by a loan officer, thus promoting accountability, as done in real-world banking scenarios. The use of SHAP and LIME helps to promote transparency by enabling applicants to understand the role of their financial features in the final decision.

**Fig. 2:**
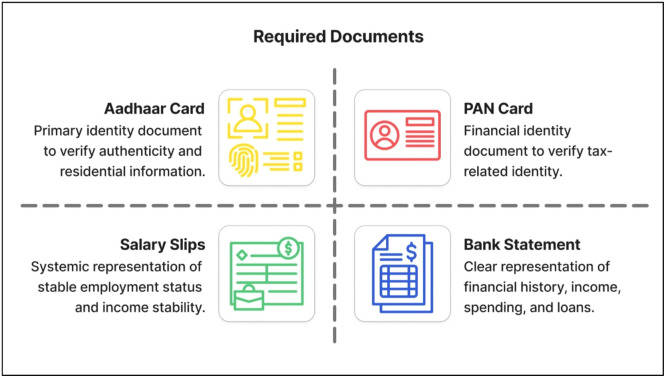
Required documents for applicant data acquisition.

The information provided in each document is unique and essential for a comprehensive assessment of the applicant’s financial credibility and authenticity of their identity. The document requirements and their roles in identity verification, income validation, and financial behavior assessment are outlined in Fig. 2.

The information extracted from the document is represented in a structured JSON format with nested properties for each document type. The extracted information can be further processed to extract features systematically.

### Data cleaning

The extracted JSON data undergoes a structured data cleaning and validation step. This step is essential because the data that is being extracted comes from a variety of financial and identity documents. It ensures that the data is accurate and suitable for feature engineering.

Normalization: Monetary values obtained from the salary slips and bank statements are standardized to a numerical format by removing the delimiters, currency symbols, and other text data. Date fields like date of birth and joining date are standardized to a particular format. In the same way, the text data like names is converted to a uniform case and whitespace is removed for easy matching.

Field Standardization and Alignment: Salary components like net pay, gross pay, and deductions; transaction values; and balances are standardized across the months. Similarly, bank transaction summaries like monthly credit, debit, EMI, and balances are aggregated to a structured format for the six months.

**Fig. 3:**
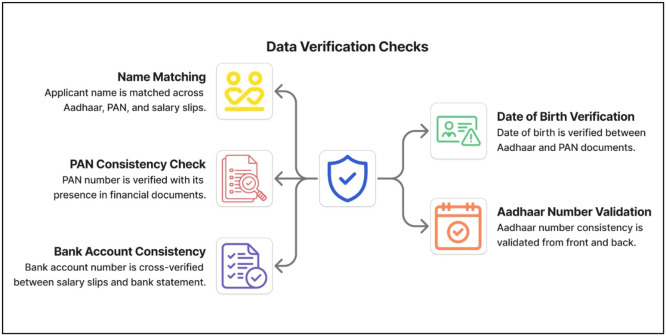
Cross-document data verification checks.

Cross-Verification: To ensure the integrity of the data, several verification checks are carried out on the data, as depicted in Fig. 3.

**Fig. 4:**
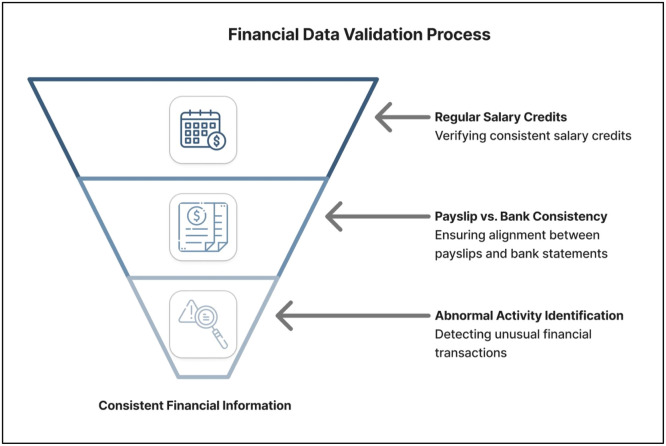
Financial data validation workflow.

Logical Consistency Checks: Further validation checks are also carried out to identify any inconsistencies in the financial information, as depicted in Fig. 4. n the event of any inconsistencies being identified during the above checks, the application is flagged for manual intervention. In this case, the applicant is required to re-upload the documents, or the loan officer intervenes to ensure the correctness of the application before feature engineering.

### Feature engineering and verification

The cleaned JSON data is transformed into a structured feature vector consisting of 15 attributes. These features are categorized as direct (extracted directly) and derived (computed from raw values). The final feature set, including both direct and derived attributes representing financial and behavioural characteristics, is defined in Table 2.

**Table 2:** Feature Description.

Feature Name	Description
income_stability	Indicates consistency of salary credits across observed months, based on regularity and variance of income inflows in bank statements
average_eligible_emi	Estimated maximum EMI repayment capacity derived as a proportion of usable income
average_usable_salary	Average disposable income after deducting existing EMI obligations from credited salary
average_monthly_credit	Mean monthly credit inflow observed in the bank account over the evaluation period
employer_category	Categorization of employer (e.g., MNC, Government, Private) indicating job reliability and income security
employment_tenure_years	Total duration of current employment in years, reflecting job stability
average_month_end_balance	Average closing balance across months, representing liquidity and financial buffer
bounce_count	Total number of failed or bounced transactions, indicating financial discipline and risk
gambling_transaction_count	Number of transactions classified as high-risk or non-essential spending behaviour
active_loans_count	Total number of ongoing loan obligations identified from bank transaction patterns
has_credit_card	Binary indicator denoting presence of credit card-related transactions or liabilities
has_personal_loan	Binary indicator denoting existence of personal loan obligations
has_home_loan	Binary indicator denoting existence of home loan obligations
credit_exposure_intensity	Ratio representing overall loan burden relative to income level
average_obligation_to_income_ratio	Average percentage of income committed toward EMI payments across months

**Fig. 5:**
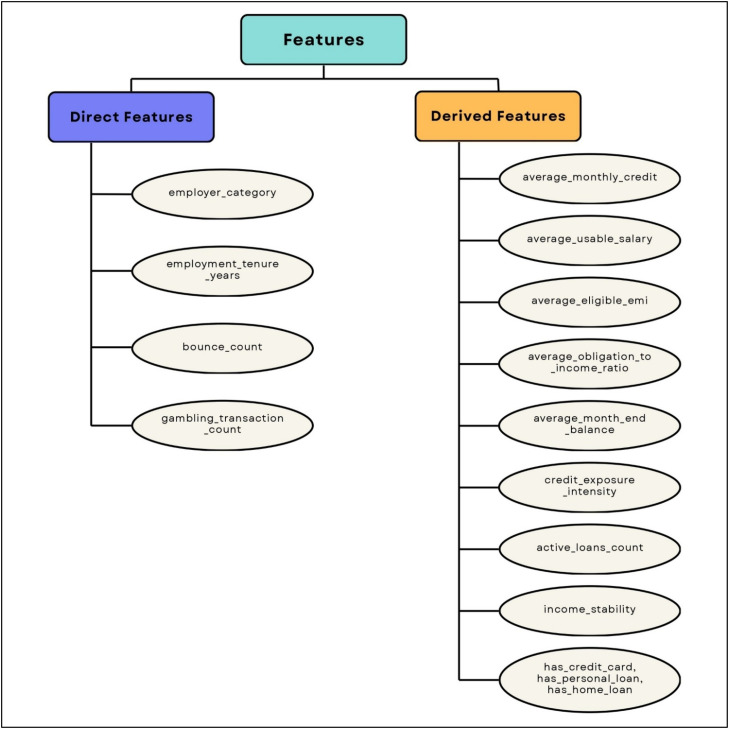
Categorization Of Financial Attributes: Direct Vs Derived Features.

As shown in Fig. 5, the feature engineering framework categorizes extracted financial attributes into direct and derived features for credit risk prediction.

Direct Features: Direct features are extracted without additional transformation:

1. employer_category: Classification of employer (e.g., MNC/Government/Private) derived from salary slips
2. employment_tenure_years: Total duration of employment computed from date of joining
3. bounce_count: Total number of transaction bounces observed in bank statements
4. gambling_transaction_count: Count of transactions categorized as high-risk spending

Derived Features: Derived features are computed from multi-month financial records obtained from salary slips and bank statements:

1. average_monthly_credit: Represents the average inflow of funds into the applicant’s account over the observation period:
  $$Average\;Monthly\;Credit=\frac{1}{N}\sum_{i=1}^{N}C_{i}$$
  where $C_{i}$ is the total credited amount in month i, and N is the number of months.
2. average_usable_salary: Captures the disposable income available after repayment obligations:
  $$Average\;Usable\;Salary=\frac{1}{N}\sum_{i=1}^{N}\left(S_{i}-EMI_{i}\right)$$
  where $S_{i}$ is the credited salary and $EMI_{i}$ is the total EMI in month i.
3. average_eligible_emi: Defines the maximum permissible EMI capacity based on income:
  AverageEligibleEMI=0.4·AverageUsableSalary
4. average_obligation_to_income_ratio: Measures the proportion of income committed toward loan repayment:
  $$OIR=\frac{1}{N}\sum_{i=1}^{N}\left(\frac{EMI_{i}}{S_{i}}\cdot 100\right)$$
5. average_month_end_balance: Reflects liquidity by averaging the closing balance across months:
  $$Average\;Month\;End\;Balance=\frac{1}{N}\sum_{i=1}^{N}B_{i}$$
  where $B_{i}$ is the closing balance for month i.
6. credit_exposure_intensity: Quantifies the applicant’s loan burden relative to income:
  $$Credit\;Exposure\;Intensity=\frac{Total\;Active\;Loans}{Average\;Monthly\;Income}$$
7. active_loans_count: Total number of active loan obligations derived from transaction categorization
8. has_credit_card, has_personal_loan, has_home_loan: Binary indicators derived from loan type identification within bank transactions
9. income_stability: Derived indicator based on consistency of salary credits across months, considering presence, frequency, and variance of income inflows

**Fig. 6:**
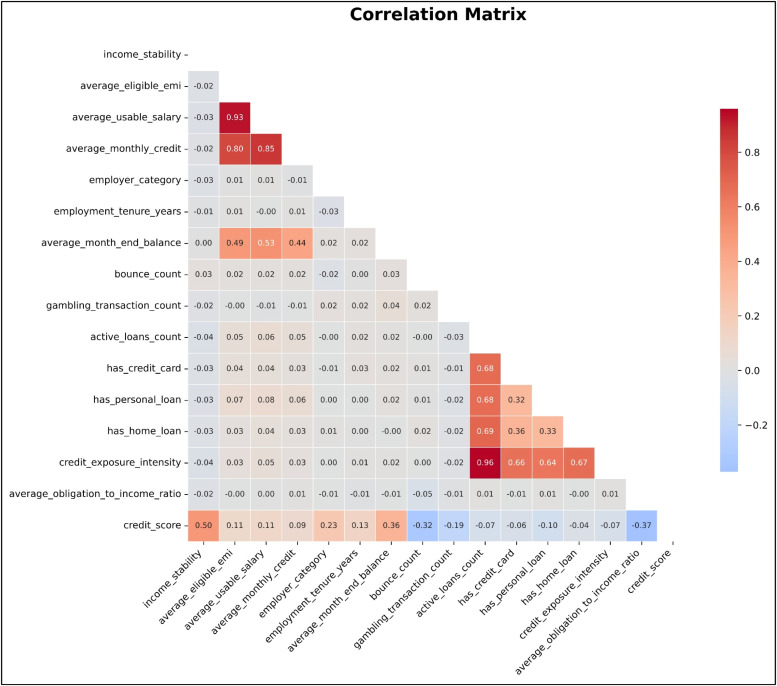
The correlation matrix of the engineered features, highlighting relationships between financial attributes and their influence on credit score.

The correlation matrix of the engineered financial features is presented in Fig. 6. Following feature generation, a human validation step is performed where the loan officer verifies the correctness of extracted and computed features before passing them to the predictive model.

### Credit risk prediction model

The predictive component utilizes the Extreme Gradient Boosting (XGBoost) algorithm, a tree-based ensemble learning method known for its effectiveness in handling structured tabular data. The model is trained using the 15 engineered features as input variables and the corresponding credit score as the target output. XGBoost iteratively builds decision trees by minimizing a regularized loss function, enabling it to capture complex non-linear relationships between financial attributes.

The input to the model is a feature vector $X=\{x_{1},x_{2},..,x_{15}\}$, where each feature represents a financial or behavioural attribute of the applicant. The model outputs a continuous numerical value corresponding to the predicted credit score:

$$\hat{y}=f\left(x\right)$$

where f(·) denotes the learned mapping function of the XGBoost model.

### Decision logic layer

The decision logic layer maps the predicted credit score to a structured form of lending decisions by applying a set rules that define loan eligibility, rate, and approved loan amounts. This layer ensures that the model prediction is mapped to consistent and policy-driven financial decision.

Initially, the predicted credit score is evaluated against a predefined threshold to determine loan eligibility:

$$Status=\left\{ \begin{array}{c} Approved,\hat{y}\geq 650\\ Rejected,\;otherwise \end{array}\right.$$

where $\hat{y}$ represents the predicted credit score. Applicants with scores below the threshold are not considered for further processing.

For approved applications, the interest rate is assigned using a tiered structure based on the predicted score, reflecting risk-based pricing. Interest Rate Structure Based on Predicted Credit Scores. The mapping between predicted credit score ranges and assigned interest rates is formalized in Table 3.

**Table 3:** Interest Rate Structure Based on Predicted Credit Scores.

Predicted Credit Score	Interest Rate (%)
$\hat{y}\geq 800$	9.5
$800>\hat{y}\geq 750$	12.5
$750>\hat{y}\geq 700$	17
$700>\hat{y}\geq 650$	24

Following interest rate assignment, the system evaluates the applicant’s repayment capacity using an Equated Monthly Instalment constraint. The EMI for a given loan amount is calculated as:

$$EMI=P\cdot r\cdot \frac{{\left(1+r\right)}^{n}}{{\left(1+r\right)}^{n}-1}$$

Where P denotes the loan principal, r is the monthly interest rate (annual rate divided by 12), and n represents the loan tenure in months.

The calculated EMI for the requested loan amount is mapped with the average eligible EMI, which was calculated as a feature. If the required EMI is found to be within the permissible limit, the requested loan amount is sanctioned. Otherwise, the maximum principal amount is recalculated by imposing the constraint of EMI within the allowable limits, thereby keeping the repayment obligation of the applicant within reasonable limits.

This decision layer employs rule-based decisions that clearly relate the predicted credit score to the final decisions regarding lending, allowing for the enforcement of financial constraints in a manner that is still aligned with the repayment capacity of the applicant.

### Explainability module

In order to promote transparency in the document-based credit score calculation, the proposed framework utilizes the SHAP and the LIME techniques to interpret the predictions made by the XGBoost model based on the 15 engineered financial features. This is because the two techniques work on the same feature vector extracted from the applicant documents, thus providing a direct connection to the extracted applicant's financial features.

SHAP is used to determine how much each feature affects a prediction. This enables the analysis of how features such as income stability and credit exposure affect the credit score. SHAP provides both global and local interpretability. It offers global interpretability by showing which features are important across all applicants and local interpretability by showing how each feature affects an individual applicant.

LIME is used to produce instance-specific explanations by approximating the model’s behaviour on the specific instance. This is done by changing the features and observing the effect on the predicted result. This helps to determine the features with the greatest influence on the decision for the specific instance.

The use of SHAP and LIME helps the system to clearly show whether the features have a positive or negative effect on the predicted score, how significant the features’ effect is on the predicted score, and which financial factors have the greatest influence on the decision. This information is used to produce an explanation report and also to produce indicative information on which financial features need to be improved to increase creditworthiness.

### Human-in-the-loop review

In order to ensure accountability and regulatory compliance, a human-in-the-loop feature is incorporated in the proposed pipeline, where the loan officer makes the final review prior to the issuance of the lending decision. In this step, the loan officer reviews the structured feature set obtained from the applicant documents, the predicted credit score obtained from the XGBoost model, and the corresponding explanation outputs obtained from the SHAP and LIME models. This step allows the loan officer to validate the data used as input and the reasoning by the model. Based on the review, the loan officer has the authority to approve or reject the loan, or override the lending decision obtained from the proposed pipeline. All the operations carried out in this step are logged, which adds to the reliability of the proposed decision-making process.

### Final output generation

The final step of the proposed pipeline is the output layer, which produces a structured output report containing the results of the predictive model, the decision logic layer, and the explainability layer. For each applicant, the system will output the predicted credit score calculated from the XGBoost model, along with the approval or rejection status of the loan application from the decision logic layer. In the case of approval, the interest rate applied to the applicant and the amount of the loan sanctioned, calculated from the EMI constraints, will be included in the output report. Furthermore, the system will produce a detailed explanation report through the use of SHAP and LIME tools to explain the results to the applicants. The output report is structured to ensure the results can be easily interpreted and made use of by the institutions as well as the applicants themselves.

## Method validation

The proposed methodology was validated using a publicly available data set obtained from the Kaggle data science repository. The data set used is structured and consists of financial and behavioural attributes related to loan applicants and is therefore applicable for credit risk prediction using supervised learning-based models. A total of 15 features were used for the data set, which includes income stability, average eligible EMI, average usable salary, average monthly credit, employer category, employment tenure in years, average month end balance, bounce count, gambling transaction count, active loans count, credit card presence, personal loan presence, home loan presence, credit exposure intensity, and average obligation to income ratio. The target variable is continuous and consists of a numerical value representing the credit score of the applicant.

The data set is highly consistent with the proposed model and is derived from a similar feature engineering pipeline that uses OCR to extract financial indicators. This indicates that the proposed model can be well generalized when tested with real-world data extracted from financial documents, hence making it possible to reproduce the proposed method.

### Dataset description

The dataset used for model validation was obtained from the Kaggle data science repository and consists of structured financial and behavioural attributes of loan applicants. The dataset contains 10,000 instances, with 15 input features and a continuous target variable representing the applicant’s credit score.

The features include a combination of numerical and categorical variables capturing income features (e.g., average monthly credit, usable salary), financial behaviour (e.g., transaction bounce count, gambling-related transactions), and credit exposure indicators (e.g., active loans, obligation-to-income ratio).

Before using these attributes for model development, the data were evaluated for integrity, consistency, and statistical distribution. There were no major missing entries, and appropriate preprocessing procedures like normalization and feature alignment were performed to make sure they are compatible with the suggested document-based feature generation pipeline.

The data were split between training and test sets on an 80:20 ratio to ensure robust model evaluation. The distribution of the target variable indicates a reasonably balanced spread of credit scores, making it suitable for supervised learning tasks.

**Fig. 7:**
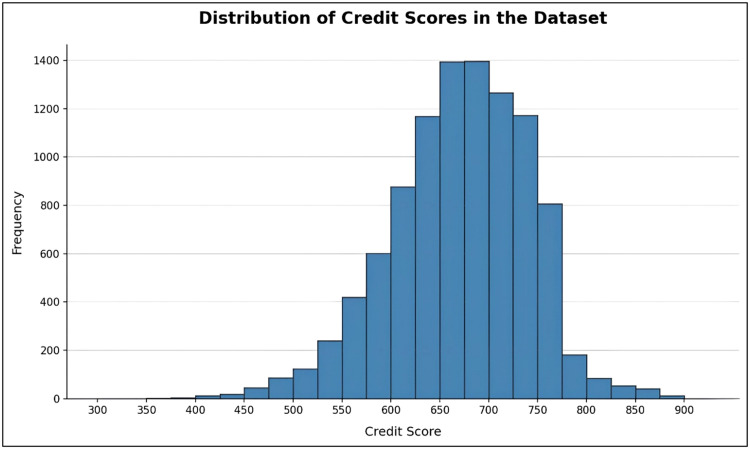
Distribution of credit score values in the dataset.

A visualization of the credit score distribution is provided in Fig. 7, illustrating the variability and range of applicant credit profiles used for model validation.

### Model evaluation

To ensure robust evaluation, the dataset was divided into training and testing subsets using an 80:20 split. All preprocessing steps, including feature normalization and alignment, were consistently applied across training and testing datasets to maintain data integrity.

The predictive performance of the regression models was evaluated using standard regression metrics.

1. Mean Absolute Error (MAE) measures the average magnitude of prediction errors:
  $$MAE=\frac{1}{N}\sum_{i=1}^{N}\left|y_{i}-{\hat{y}}_{i}\right|$$
2. Root Mean Squared Error (RMSE) measures the square root of the average squared prediction errors, giving higher weight to large errors:
  $$RMSE=\sqrt{\frac{1}{N}\sum_{i=1}^{N}{\left(y_{i}-{\hat{y}}_{i}\right)}^{2}}$$
3. Coefficient of Determination (R² Score) represents the proportion of variance in the dependent variable explained by the model:
  $$R^{2}=1-\frac{\sum {\left(y_{i}-{\hat{y}}_{i}\right)}^{2}}{\sum {\left(y_{i}-\bar{y}\right)}^{2}}$$
  For decision-level evaluation, the regression outputs were converted into binary outcomes based on a predefined threshold.
4. Accuracy measures the proportion of correctly classified instances:
  $$Accuracy=\frac{TP+TN}{TP+TN+FP+FN}$$
5. Precision measures the proportion of correctly predicted positive instances among all predicted positives:
  $$Precision=\frac{TP}{TP+FP}$$
6. Recall measures the proportion of correctly predicted positive instances among all actual positives:
  $$Recall=\frac{TP}{TP+FN}$$
7. F1 Score represents the harmonic mean of Precision and Recall:
  $$F1=2\cdot \frac{Precision\cdot Recall}{Precision+Recall}$$

These metrics collectively provide a comprehensive assessment of prediction accuracy, error distribution, model fit, and decision-level reliability.

### Performance comparison

The performance of the proposed XGBoost model was compared to LightGBM, Random Forest, Logistic Regression, and Long Short-Term Memory (LSTM) models to assess the traditional and state-of-the-art learning techniques on structured financial data. Table 4 shows the regression performance comparison for all the models.

**Table 4:** Model Performance Comparison.

Model	MAE	RMSE	R² Score
LightGBM	14.05	19.03	0.9261
LSTM	16.42	20.70	0.9018
Random Forest	18.95	22.41	0.8824
Logistic Regression	24.60	26.28	0.8427
XGBoost (Proposed)	13.71	18.87	0.9243

**Fig. 8:**
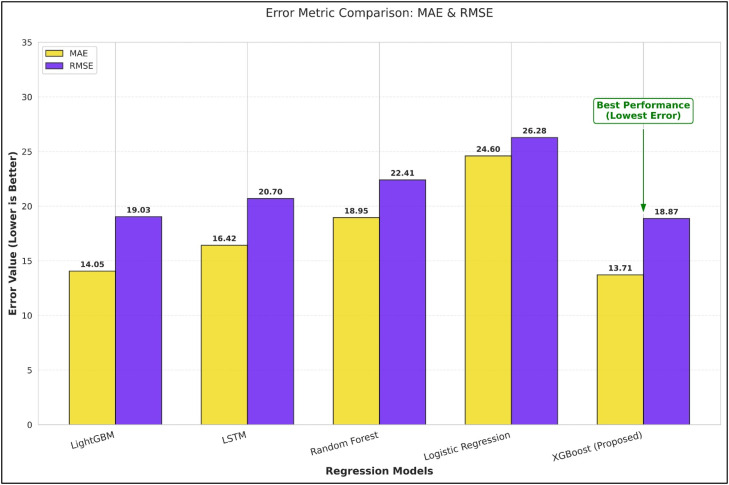
Regression performance comparison across models based on MAE and RMSE.

From the results, it is evident that XGBoost has the lowest values for error calculation, i.e., MAE and RMSE. This shows the high consistency in prediction. Although LightGBM has a slightly higher R² score, XGBoost performs better in the overall error minimization criteria. Therefore, XGBoost is more appropriate for the prediction of financial risk. Fig. 8 compares regression performance across models based on MAE and RMSE, highlighting differences in prediction error.

At the decision level, the predicted credit score is used to make a binary classification using a threshold value of 650. This facilitates the classification of loan applications into approval or rejection. The performance evaluation is done using Accuracy, Precision, Recall, and F1 Score. Table 5 presents the performance comparison at the decision level.

**Table 5:** Decision-Level Performance.

Model	Accuracy	Precision	Recall	F1-Score
LightGBM	0.908	0.912	0.924	0.918
LSTM	0.81	0.68	0.33	0.44
Random Forest	0.87	0.74	0.59	0.66
Logistic Regression	0.91	0.89	0.87	0.88
XGBoost (Proposed)	0.925	0.938	0.945	0.942

**Fig. 9:**
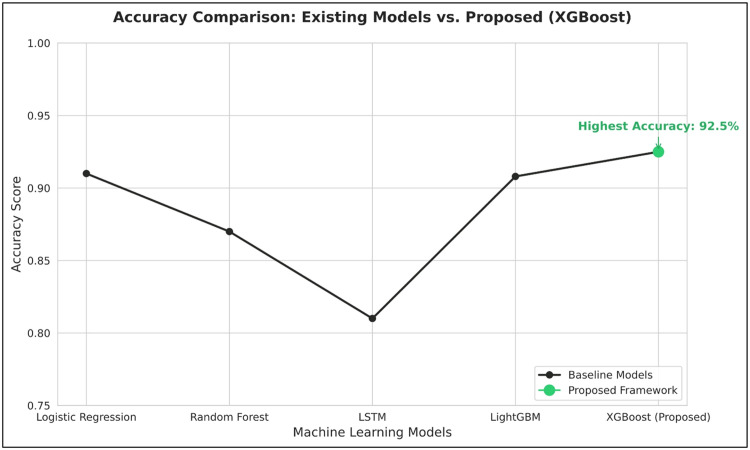
Classification performance comparison across models based on accuracy.

**Fig. 10:**
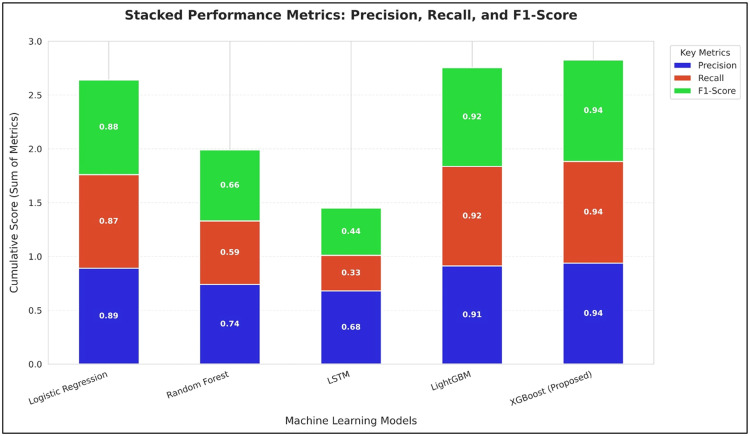
Classification performance comparison across models based on precision, recall, and F1-score.

The results of the classification further verify the effectiveness of XGBoost, as it has the highest accuracy as well as precision-recall performance. This shows that the regression results are correctly mapped to the decision results by the rule-based thresholding mechanism. Fig. 9, Fig. 10 show the comparison of classification performance across all models.

### Explainability analysis

In order to provide a level of transparency and interpretability for the proposed credit scoring model, explainability analysis was conducted using both the SHAP and LIME methods. These approaches provide a level of global and local interpretability for the XGBoost model, utilizing the same 15 engineered financial features from the document processing pipeline.

**Fig. 11:**
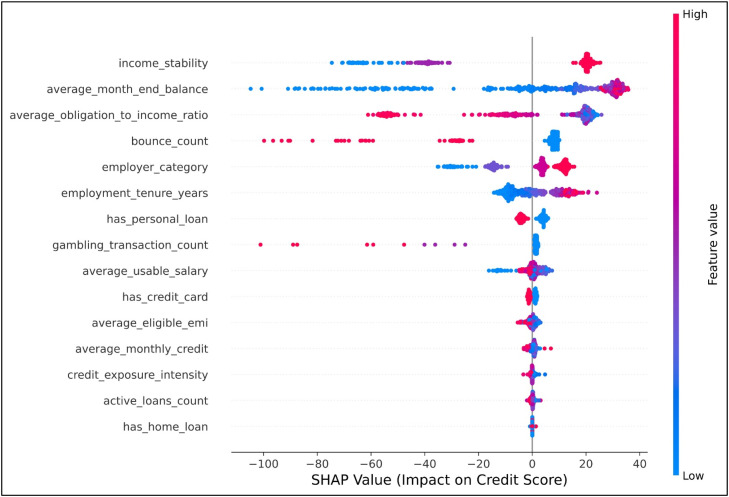
SHAP-based global feature importance illustrating feature contributions across all instances.

Fig. 11 presents a SHAP beeswarm plot illustrating global feature importance and the contribution of each feature to the predicted credit score. The distribution of the SHAP values across all instances shows that income_stability, average_month_end_balance, and average_monthly_credit have a positive contribution towards a higher credit score, which represents high financial consistency and liquidity. On the other hand, features like bounce_count and average_obligation_to_income_ratio have a high negative contribution towards the predicted credit score, which represents high financial risk. This consistency in the relationship with the principles of financial risk assessment provides a high degree of reliability for the feature engineering.

**Fig. 12:**
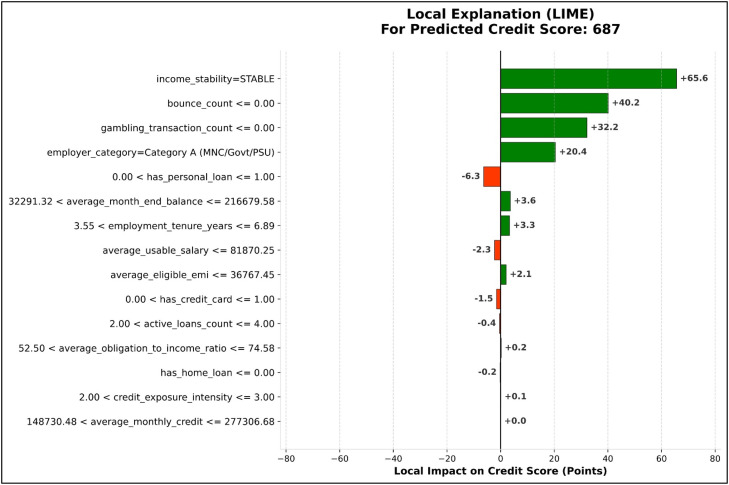
LIME-based local explanation illustrating feature contributions for an individual prediction.

In addition to the global interpretability results, local explanations were also generated to explain the predictions made by the model individually. Fig. 12 shows local explanation results generated using the LIME method for predictions made on an applicant. The local explanations highlight the features that have the highest impact on the predictions made by the model. LIME is a local interpretability technique that approximates model behaviour around a specific instance to generate interpretable explanations. It identifies the features that have the most significant influence on individual predictions.

The results obtained through the LIME method match the results obtained through the SHAP method, which is a clear indication of the consistency of the model interpretability framework. The results show that the model is interpretable at the macro as well as the micro level through the use of the SHAP and LIME model interpretability methods.

While SHAP and LIME provide quantitative insights into feature contributions, their practical relevance lies in supporting real-world credit decision-making.

For instance, consider an applicant with a predicted credit score of 687, which lies near the approval threshold of 650 defined in the decision logic layer. The SHAP analysis indicates that features such as income stability, average monthly credit, and account balance contribute positively to the score, reflecting consistent financial behaviour. However, features such as a high obligation-to-income ratio and multiple active loans contribute negatively, indicating increased financial burden.

From a business perspective, this interpretation enables the loan officer to justify the decision transparently. Although the applicant may qualify for approval, the identified risk factors may lead to adjustments in lending terms, such as assigning a higher interest rate or reducing the sanctioned loan amount to maintain repayment feasibility.

Similarly, LIME-based local explanations for the same instance highlight that repayment-related features have the strongest influence on the prediction. This provides instance-level clarity, allowing the decision to be explained to the applicant in an understandable manner.

Furthermore, these explanations can be used to provide actionable feedback. For example, reducing existing loan obligations or improving income consistency can positively influence the credit score, thereby improving eligibility in future applications.

This demonstrates that the proposed framework not only enhances prediction accuracy but also enables transparent, interpretable, and actionable credit decision-making in real-world financial systems.

### Model efficiency and observations

The comparative evaluation of the models has revealed that XGBoost has the best balanced performance in terms of prediction accuracy, error minimization, and stability when the structured financial features are extracted based on the input provided in the form of documents. Table 4 indicates that XGBoost achieves the lowest MAE and RMSE, indicating its consistency in terms of predictions, having the least deviation from the actual credit score. Fig. 8 illustrates that the error value for XGBoost has the least value among all the models. This consistency is critical in the context of the credit score, as the prediction error may have a major impact on the final decision regarding the approval, interest rate, and loan amount.

In contrast, LightGBM demonstrates a performance close to that of XGBoost, as both models are gradient boosting-based and can handle non-linear relationships between financial attributes [26]. However, slightly higher error value readings for LightGBM may imply a degree of variability in prediction stability. The performance of Random Forest can be considered robust due to its ability to average predictions. However, it demonstrates higher error value readings compared to other models, which may imply a lack of ability to handle complex relationships between features like income stability, repayment ability, and credit exposure. The lowest performance is demonstrated by Logistic Regression model, which reconfirms that linear models are not capable of handling complex financial behaviour represented by engineered features.

The addition of the LSTM module allows the evaluation of the effectiveness of deep learning-based solutions for this particular scenario; nonetheless, the effectiveness of the latter still remains lower compared to tree-based ensemble models [27]. This is perhaps due to the nature of the input data, which is aggregated in the form of tabular features. Thus, the above observations conclude that boosting-based models remain more appropriate for structured financial datasets that have been generated through feature engineering-based pipelines. In summary, the above observations have reconfirmed the effectiveness of the XGBoost model not only in terms of prediction efficiency but also in terms of its integration with explainability-based models like SHAP and LIME for the development of explainable credit scoring systems driven by documents [28].

## Limitations

The primary limitation of the proposed method is its dependency on the accuracy of Optical Character Recognition and document parsing, which might vary with document quality, document type, and noise level in the document.

Furthermore, the proposed model is trained on a structured dataset, which might not cover all possible variations and diversity of actual financial data, considering various heterogeneous sources of documents.

Moreover, though SHAP and LIME help in better interpretability of the model, they might not cover all possible interactions of the model, which might hinder a deeper level of explanation of the model's output.

## Ethics statements

Not applicable.

## CRediT author statement

SM, HP, AN, SN, ON: Conceptualization. HP, SN, ON: Formal analysis, Methodology, Project administration. HP, ON, SN, AN: Resources, Data curation. HP: Writing- Original draft preparation. SM, BK: Investigation, Supervision. HP, SN, ON, AN: Validation. SM, BK, HP, ON, SN, AN: Writing- Reviewing and Editing. SM,BK: Funding acquisition.

## Declaration of competing interest

The authors declare that they have no known competing financial interests or personal relationships that could have appeared to influence the work reported in this paper.

## Data Availability

I have shared a Link to my data:
